# Introducing Virtual Points in Equivariant Networks by Extending Atom Representation for Effective Prediction

**DOI:** 10.1021/acs.jctc.5c00701

**Published:** 2025-08-25

**Authors:** Bumju Kwak, Jeonghee Jo

**Affiliations:** † Independent researcher, Seoul 06611, Republic of Korea; ‡ Independent researcher, Seoul 07795, Republic of Korea

## Abstract

Recent equivariant models embed a molecule as a set of atoms fixed in three-dimensional space, which is analogous to a ball-and-stick view. This perspective provides a concise view of molecular configurations; however, these representations may be limited in incorporating the surrounding environments of atomic nuclei, including electron configurations. To overcome this limitation, we propose neural polarization (NP), a novel method that extends equivariant networks by embedding each atom as a pair of an atom and virtual points. Motivated by electron density configurations, NP represents each atom as a pair comprising the original fixed atom and a virtual atom whose position is updated through parameterization during model training. NP can be flexibly applied to most types of existing equivariant models. We showed that NP can improve the prediction performance of existing models over a wide range of targets, including electron density. Our experimental results on various benchmarks suggest new insights, indicating that the extended atomic representations can improve the overall molecular tasks.

## Graphical abstract

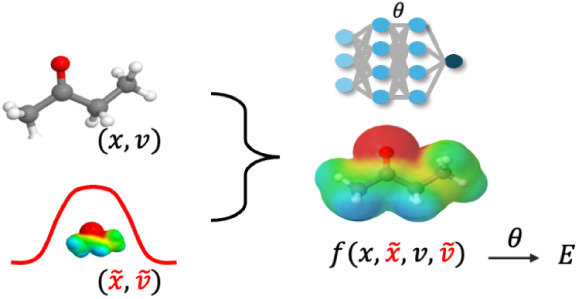

## Introduction

Density functional theory (DFT) has been a fundamental methodology in classical computational chemistry,^1–4^ providing reliable approximations of molecular configurations. From the perspective of DFT, the main strategy for solving the Schrödinger equation of a molecule is to consider the many-electron system as a unique functional for a single function of the electron density of the molecule, rather than an *N* number of wave functions.^5,6^

Recent advances in artificial intelligence have provided an alternative approach through deep neural networks that can model molecular systems while significantly improving computational speed, thereby increasingly contributing to enhanced efficiency in molecular simulation research.^7–12^ The field of machine learning (ML)-based approaches for DFT calculations has usually been developed for the prediction of the energy-related properties of molecules, driven by the need to reduce computational costs in DFT calculations. Many of these studies used molecular configurations or atomic coordinates to predict the energies calculated by DFT, with several initial cases introduced as follows: Montavon *et al*.^13^ first developed the ML-based modeling of atomization energies including HOMO and LUMO of small organic molecules, based on the quantum mechanical principles. Lee et al.^14^ and Pereira et al.^15^ also established ML-based regression approach for predicting band gaps of inorganic compounds. Chandrasekara et al.^16^ applied deep learning on Molecular Dynamics (MD) simulations.

Building on these previous studies, public benchmarks for quantum mechanical properties of various molecules were introduced, such as QM9 ^17^ and MD17.^18,19^ Deep neural networks that learn atomic features based on message passing have shown strong performances on these benchmarks.^20,21^ This facilitated the development of more effective and domain-driven approaches for realistic and accurate representation of molecular structures, for example, rotation-equivariant graph neural networks (EGNN^22^ and TorchMD-NET)^23^ and transformer-based models (Equiformer)^24^ represented in 3D Euclidean space. With advancements in predicting molecular properties, more sophisticated and challenging tasks have been introduced to the field. These include energy-related properties of excited-state molecules (xxMD),^25^ where several models have achieved prediction performance comparable to DFT-level calculations. These efforts also led to the emergence of molecular charge density data sets, accompanied by models designed to predict electron density values on three-dimensional grids encompassing molecular structures.^26,27^

Despite these improvements, traditional message passing for molecules based on single-hop and atom-centric updates remained a key challenge, limiting the models’ ability to fully capture complex molecular interactions. To overcome these limitations, researchers have developed two types of approaches. The first approach is a virtual node adaptation to enhance GNNs by introducing artificial nodes to incorporate more global patterns of molecules, which are not limited to representations of existing atoms. For example, Li et al.^28^ introduced neural atoms that effectively reduce arbitrary node-pair interactions to single-hop communications, significantly improving prediction performance by capturing long-range interactions inside molecules. Okabe et al.^29^ demonstrated that the phonon prediction performance of crystals can be improved using virtual nodes without additional domain-driven knowledge. Last, Sestak et al.^30^ also showed that introducing an artificial node in a molecule can both improve performance and learn to represent specific molecular features in the binding site identification task.

The second approach directly encodes the surrounding atomic environments through physics-based representations. Zhang et al.^31^ proposed the Embedded Atom Neural Network (EANN), which reported high accuracy with fewer parameters by implicitly capturing multibody interactions based on density vectors. Building on this, Zhang and Jiang^32^ developed FIREANN, which incorporates field-dependent features into atomic descriptors to represent system-field interactions with rotational equivariance. This approach successfully correlates various response properties with potential energy in a single model, demonstrating the effectiveness of embedding environmental information directly into atomic representations.

Complementary to these approaches, several deep neural networks have been developed to directly consider the density functional of chemicals for advanced electron-based molecular tasks. Brockherde et al.^33^ overcame the derivative calculation challenge by directly learning density potential and energy density maps, successfully performing the first molecular dynamics simulation capturing intramolecular proton transfer. Similarly, Chandrasekaran et al.^16^ developed a grid-based approach to predict electronic charge density distributions for diverse hydrocarbons, achieving accurate and transferable predictions across various molecular systems. Kamal et al.^34^ developed a robust charge density prediction model for diverse hydrocarbons using deep neural networks and grid-based fingerprints, training on billions of data points to map atomic neighborhoods to reference density distributions. Their model showed excellent accuracy and transferability, suggesting that machine learning can effectively predict charge densities across various material systems. Further advancing this field, DeepDFT^26,35^ emerged as one of the remarkable benchmarks for direct training of density functionals, demonstrating significant improvements in computational efficiency while maintaining accuracy. These developments highlight the growing recognition in the deep learning community of the importance of learning DFT-based properties directly from data.

Despite the crucial role of electron density distribution in molecular property analysis, previous deep neural networks for molecular property prediction^24,36–44^ may be potentially limited due to the exclusion of effects from atom surroundings. Considering these previous research findings and the underlying principles of DFT as domain knowledge, we assumed that approaches predicting quantum mechanical properties based solely on atomic positions have fundamental limitations.

To address these limitations with a more comprehensive representation of molecules, we propose a novel flexible extension method for rotation-equivariant networks motivated by DFT: neural polarization (NP). This method allows each equivariant block to explicitly consider the atom surroundings while maintaining equivariance. The key point of NP is the introduction of an additional virtual point (VP) for each atom, which can update its location during the training process in an unsupervised way. These VPs can be viewed as a type of feature descriptor of atom surroundings outside the atom centers. Our methodology differentiates itself from existing virtual point approaches in the following ways. While other works typically assume a single supernode or a small number of nodes per molecule, not specifically aimed at describing atomwise environments, our approach introduces 3D rotation-equivariant virtual points for each atom to describe their environments in 3D Euclidean space. We hypothesize that this method better captures the principles of electron density effects determined by individual atomic influences, ultimately leading to superior molecular representation learning.

We applied NP to four existing equivariant networks for quantum mechanical property prediction and trained the enhanced models from scratch. We verified that NP significantly improved the prediction performance over a wide range of prediction targets, including electron density, compared to the original reports. The visualization of experimental results in the electron density prediction task revealed that our approach can significantly improve the model’s performance, especially in molecules occupied by delocalized electron density.

Besides reporting prediction performances on the benchmarks, we also conducted various ablation studies to verify the effectiveness of NP. From these experimental results, we concluded that the prediction improvement of NP did not come from just higher computational costs or duplicating atom points but the availability of updating positions of virtual points.

The contributions of this study are summarized as follows.

Motivated by DFT, we developed a novel extension method, NP, for equivariant networks by introducing VPs expecting that they can incorporate delocalized electron density for advanced molecular representation.
We validated the effectiveness of NP on various types of equivariant networks over a wide range of prediction targets. In addition, we found that NP improves prediction performance, especially at the delocalized electron density region in molecules.
We verified that NP reduces the approximation error with the original network and improves the expressive power of the baseline model.

## Methods

### Equivariant Neural Networks

By definition, a group-equivariant network consists of group-equivariant layers such that its output transforms equivariantly under specified group operations applied to its input.^45^ A *G*-equivariant layer is a function of *f* which satisfies *g* ◦ *f*(*x*) = *f*(*g* ◦ *x*) for any group element *g* ∈ *G*. For representing molecular structures in Euclidean space, an orthogonal geometry group SO(3) or SE(3) is generally selected as *G* in many message passing-based deep neural networks.^36,37,40–42^ For predicting a molecular property *y* related to its energy for learning molecular embedding representation *E*(**x**) from atom position **x** = {*x*_*i*_}, the network of *T* layers should be group invariant, consisting of 0,···,*T* – 1 group-equivariant layers *M* with the final *G*-invariant readout function *R* or a pooling layer. That is, *Ŷ* = *R* ◦ *M*_*T*‑1_ ◦ *M*_*T*‑1_ ◦··· ◦ *M*_0_ ◦ *E*(**x**). All mathematical notation used throughout this manuscript is defined in Table 1.

**Table 1:** Table of Notations in This Manuscript

Variable	Definition
*i*	an index of an atom of a molecule
*t*	a layer index of a baseline network
*N*	the number of atoms in a molecule
*x*_*i*_	a three-dimensional coordinate of *i*-th atom
*a*_*i*_	an atom number (or atom type) of *i*-th atom
*v*_*t*,*i*_	a node feature of *t*-th Layer output
**x**	a set of {*x*_0_, *x*_1_, ..., *x*_*N*‑1_}
*ŷ*	predicted target (molecular property)
*X*	molecule conformation
ρ	electron density

**Embedding layer**. The embedding layer learns a feature vector of individual atoms, **v**_0_ = {*v*_*i*_} from atom positions, **x** = {*x*_*i*_} and atom numbers, **a** = {*a*_*i*_}.

**Equivariant layer**. We denote that *M*_*t*_ uses the given position **x** and the learnable equivariant vector **v**_**t**_; however, some networks only use **v**_**t**_ for learning **v**_t + 1_.

**Pooling (readout) layer**. A pooling layer *R,* located at the end of a network, also called a readout function, produces the output *ŷ*.

1 $$Embedding\;layer:v_{0}=E\left(x,a\right)$$

2 $$Equivariant\;layer:v_{t+1}=M_{t}\left(x,v_{t}\right)$$

3 $$Pooling\;layer:ŷ=R\left(v_{T}\right)$$

### Motivation for Neural Polarization

Our methodology is inspired by the polarization basis sets used in DFT, which enrich the basis functions to capture asymmetric distortions in electron density. DFT provides a framework for determining molecular properties from atomic coordinates via the electron density.^46^ Typically, the Kohn–Sham equations^47^ are solved to obtain the electron density, and subsequently, density functionals are applied to compute molecular properties. The Hohenberg–Kohn theorem^48^ establishes that the ground-state electron density uniquely determines all ground-state properties.

Correspondingly, standard equivariant neural networks learn latent features from atomic positions and their corresponding feature vectors (*x*_*i*_, *v*_*i*_) and use these features to predict molecular properties. Motivated by this analogy and the Hohenberg–Kohn theorem, we hypothesize that enriching the latent features to better capture characteristics related to the electron density may improve prediction accuracy.

Among the characteristics considered for electron density, we focused on the term polarization. Polarization refers to the asymmetric distortion of an atom’s electron cloud due to external fields or interactions with neighboring atoms. In DFT, polarization is accounted for by using high-angular momentum polarization functions that extend the basis set, enabling a more flexible description of electron density—especially its deviation from spherical symmetry around atoms. However, existing graph neural networks employ message representations that are inherently atom centered, which may limit their ability to capture broader-scale effects, such as polarization effects. Addressing these broader-scale effects in message passing neural networks remains a significant challenge, and our work specifically targets the fixed-center message constraints present in existing equivariant networks.

Drawing inspiration from the functional role of polarization basis functions in DFT, we propose a new method that extends an equivariant network by incorporating pairs of virtual positions and features—denoted (*x̃̃*_*i*,_ *ṽ*_*i*_), or virtual points (VPs)—into the latent feature representation. Just as polarization basis functions enhance the DFT basis set to better represent electron density, we expect that our extended representations will enable the network to model polarization effects more effectively. The overall framework, based on our motivation, is presented in Figure 1.

**Figure 1:**
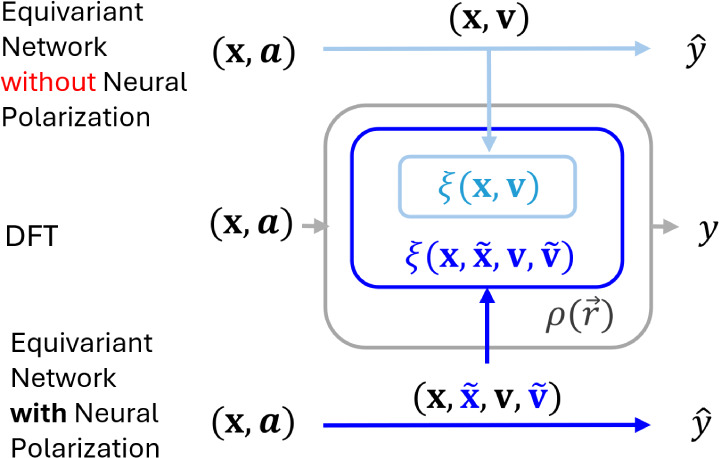
Conceptual overview of our research compared to other methodologies. In DFT, there exists a one-to-one correspondence between ρ­(*r⃗*) and molecular conformation {**x**, **a**}, which fully determines the other properties of the molecule. The baseline networks can provide a richer representation of electron density ρ­(*r⃗*) with NP.

Specifically, the proposed extended atomic positions and feature vectors (*x*_*i*_, *v*_*i*_) are augmented with additional pairs (*x̃̃*_*i*_, *ṽ*_*i*_). These VPs are obtained via a learnable equivariant transformation conditioned on the local atomic environment. Our proposition is based on the hypothesis that the learnability of these virtual points provides additional degrees of freedom beyond fixed atomic positions, enabling the model to efficiently incorporate the feature of the delocalized nature of electron density, which is a key factor in determining molecular properties. We suggest that VPs introduce additional degrees of freedom that serve a similar purpose: enhancing the expressivity of the latent representation. We expect that this improved capacity to model polarization effects will lead to better performance across a wide range of chemical tasks compared to networks relying solely on standard atom-centered features.

### Neural Polarization

We present a detailed equivariant neural network architecture enhanced with neural polarization (NP) in Figure 2. The first step is the initialization of the VPs. Given the original atom positions $x\in R^{N\times 3}$ and atomic number **ã**_0_, the initial VP positions x̃_0_ are set equal to the atom positions **x**. An embedding layer *E* then creates the initial VP features ${ṽ}_{0}\in V^{N\times k}$ . Here, $V^{N\times k}$ denotes a tensor holding *k*-dimensional features for *N* particles, where the feature space V can represent equivariant features depending on the baseline network.

4 $${\mathrm{x̃}}_{0}=x$$

5 $${ṽ}_{0}=E\left({\mathrm{x̃}}_{0},ã\right)$$

**Figure 2:**
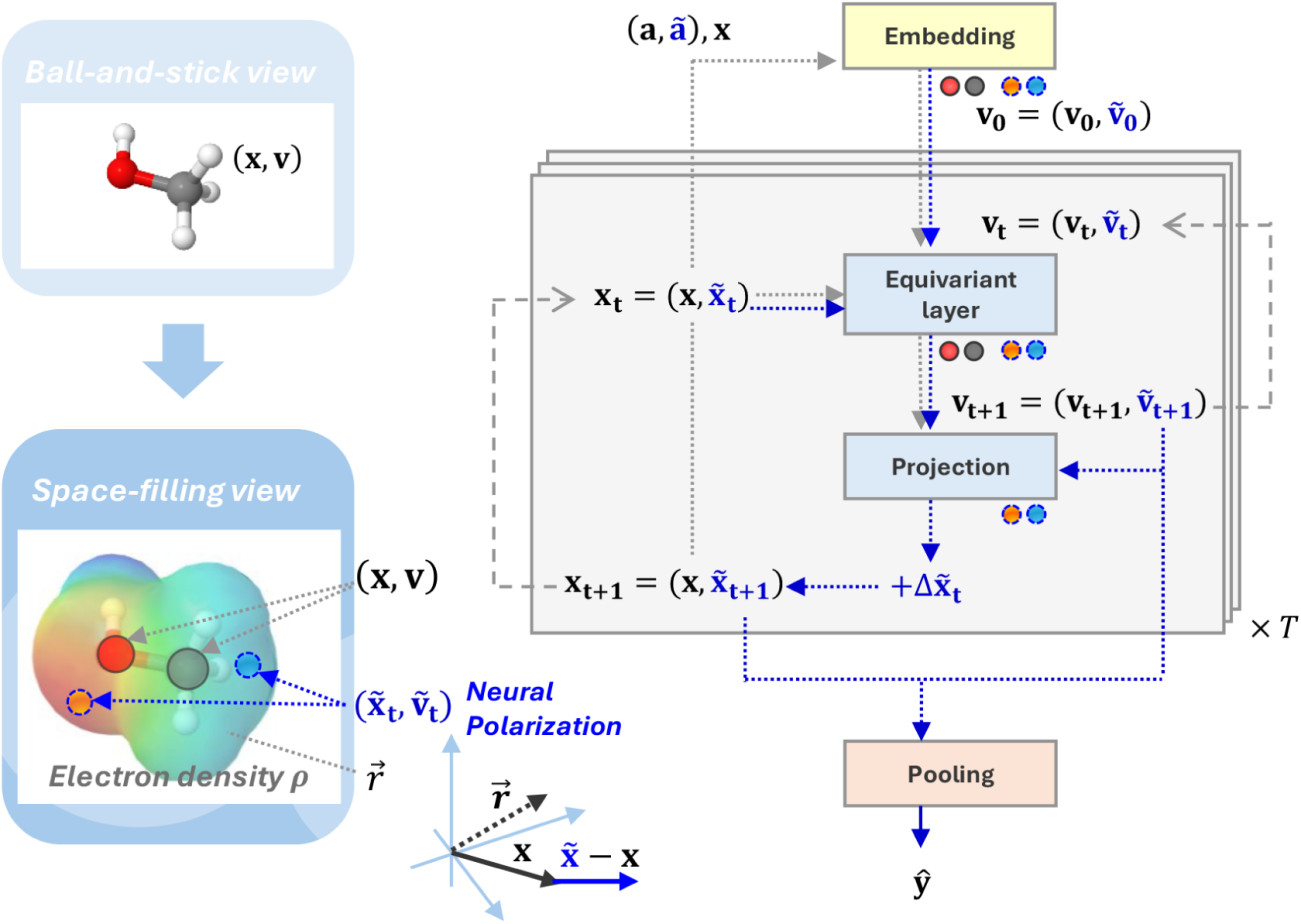
Comparison between ball-and-stick view and the space-filling view (left) and the overall architecture of NP-enhanced equivariant networks (right). NP allows a molecular representation to incorporate a nearby environment, including polarization, which is more analogous to a space-filling view of a molecule. We expect that two terms, **x̃** and **ṽ**, can infer and utilize the characteristics of an electron density ρ of each molecule during the training process, without any additional information or constraints.

Second, the network updates features by using an update layer. Since the original update layer *M*_*t*_(**x**, **v**_*t*_) in equivariant networks typically handles an arbitrary number of nodes, we employ the exact same operations as *M*_*t*_, except that layer is applied to the combined system of atoms and VPs. The input uses concatenated positions [**x**;**x̃**_*t*_] and features [**v**_*t*_;**ṽ**_*t*_]. The operator [*A*; *B*] stacks tensors *A* (for atoms) and *B* (for VPs) along the node dimension, resulting in a single tensor encompassing all 2*N* particles (*N* atoms + *N* VPs).

6 $$\left[v_{t+1};{ṽ}_{t+1}\right]=M_{t}\left(\left[x;{\mathrm{x̃}}_{t}\right],\left[v_{t};{ṽ}_{t}\right]\right)$$

Last, the VP positions **x̃**_*t* + 1_ are updated by an additional, learnable projection layer $\pi_{t}:V^{N\times k}\to R^{N\times 3}$ . We typically construct π_*t*_ as a simple sequential block consisting of an equivariant layer followed by a linear layer that transforms features into 3D vectors, but other equivariant architectures for π_*t*_ are also possible. The VP positions are then updated residually. Note that the original atom positions **x** remain fixed throughout the layers; only the VP positions **x̃**_*t*_ are modified.

7 $$\Delta {\mathrm{x̃}}_{t}=\pi_{t}\left({ṽ}_{t+1}\right)$$

8 $${\mathrm{x̃}}_{t+1}={\mathrm{x̃}}_{t}+\Delta {\mathrm{x̃}}_{t}$$

Note that although NP increases computational operations by processing 2*N* atoms, the shared weight parameters between *x* and *x̃* limit the parameter overhead to the embedding layer expansion and the addition of projection layers, rather than a full doubling of the model parameters. The pseudocode of NP is described in Algorithm 2, compared with the original framework in Algorithm 1.
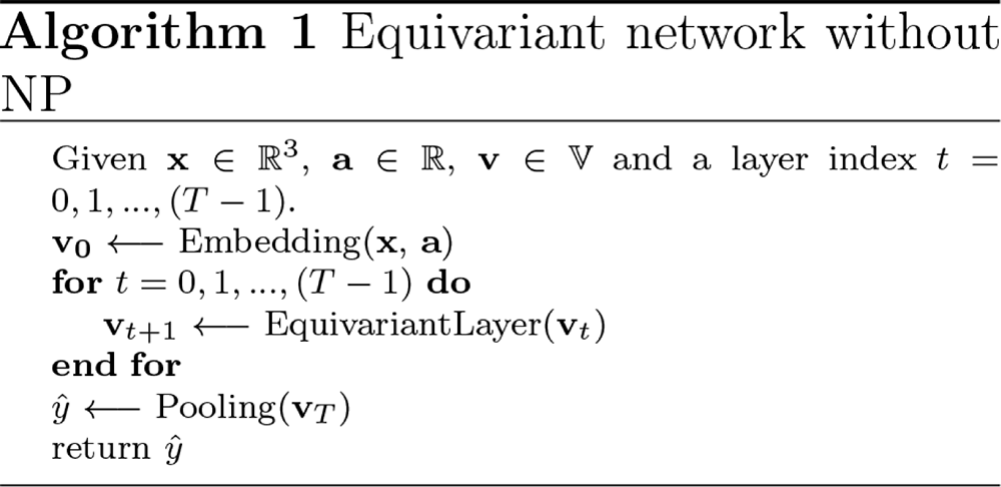
 
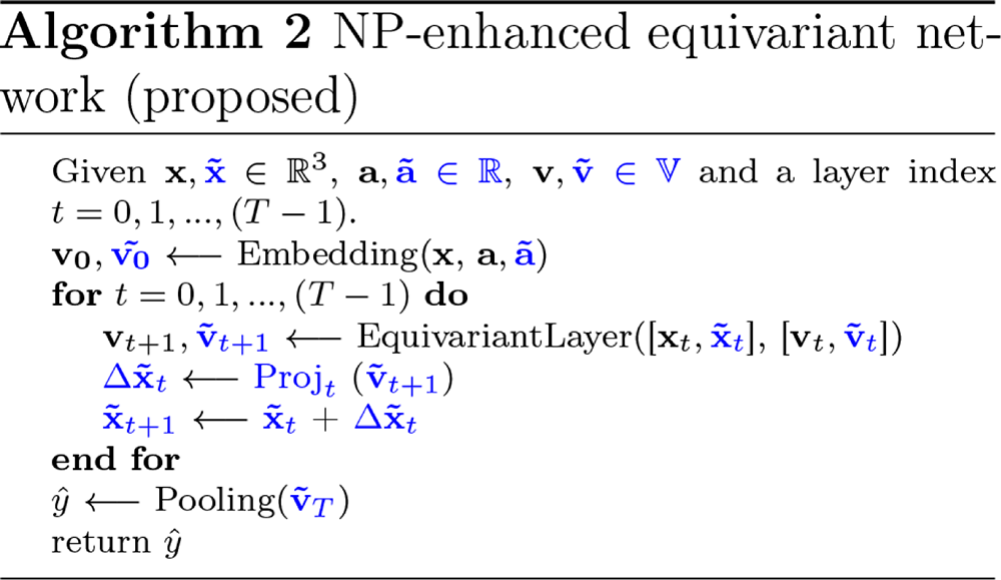


## Experiments

We evaluated the efficacy of NP across various prediction tasks by using several equivariant models. In this section, we introduce results from four types of experiments: electron density prediction (QM9 density data set,^26^ excited-state molecule property prediction (xxMD),^25^ QM9 property prediction tasks,^17^ and revised MD17 energy and force prediction tasks.^49^ Four equivariant neural networks (EGNN,^22^ DeepDFT,^26,35^ which is based on the PaiNN^40^ architecture, Equiformer,^24^ and TorchMD-NET^23^) were selected as our baseline architectures in these experiments. We followed official implementations[Fn fn1] and their train-test splits for all baseline models. In addition, we investigated NP’s effect on learned molecular features, visualized the predicted electron density plots, compared them with those obtained from the original models, and analyzed the results to highlight the advantages of our methodology. We also present training curves from different data sets in Figure S5. Further experimental details, including data sets, implementation details, and ablation studies, are provided in each section of the Supporting Information.

### Performance Gains on QM9 and MD17 DataSet

To validate our hypothesis that incorporating NP can benefit diverse molecular property prediction tasks, we conducted experiments on the QM9 and MD17 data sets, employing a range of established models.

The experimental results on the QM9 data set are presented in Table 2, categorized by whether NP was applied (marked as “with NP”) or not for each baseline network in all 12 target cases. Most of the baseline results (on the left side) of each previous model were reproduced by training the source code provided in the official repository from scratch. A few results were taken from the original papers due to computational or compatibility issues with the provided source code.

**Table 2:** MAE of the Energy and Forces Prediction on the QM9 Data Set^a^

		EGNN		TorchMD-NET		Equiformer		
Target	Unit	w/o NP	w/NP	w/o NP	w/NP	w/o NP	w/NP	avg. Δ%
μ	D	**0.029**	0.03	**0.011**	0.014	0.011	**0.010**	+4.92%
α	${a_{0}}^{3}$	**0.071**	**0.071**	0.059	**0.0447**	**0.046**	0.0527	**–3.31%**
ϵ_HOMO_	meV	**29**	29.9	20.3	**18.4**	**16.5**	16.7	**–2.04%**
ϵ_LUMO_	meV	25	**23.4**	**17.5**	17.8	14.3	**14.0**	**–2.43%**
Δ_ϵ_	meV	48	**47.9**	**36.1**	41.9	**30**	33.7	+8.20%
<*R* ^2^>	${a_{0}}^{2}$	0.106	**0.089**	**0.033**	0.085	0.251	**0.162**	**–4.29%**
*zpve*	meV	1.55	**1.50**	1.84	**1.22**	**1.26**	2.15	**–4.25%**
*U*_0_	meV	11	**9.52**	6.15	**5.5**	6.59	**5.54**	**–15.44%**
*U*	meV	12	**10.33**	6.38	**5.4**	6.74	**5.49**	**–19.03%**
*H*	meV	12	**9.52**	6.16	**5.6**	6.63	**6.27**	**–13.93%**
*G*	meV	12	**11.5**	7.62	**6.6**	7.63	**7.01**	**–9.55%**
*C*_*v*_	cal/(mol K)	**0.031**	0.032	0.026	**0.023**	**0.023**	**0.023**	**–3.31%**
	avg. Δ%		**–6.84%**		**–5.29%**		**–4.76%**	**–5.63%**

a EGNN, TorchMD-NET, and Equiformer were selected as the baseline models.

When enhanced by NP, EGNN showed error reduction for 9 targets with an average of −6.84%, TorchMD-NET showed reduction in 7 targets with an average of −5.29%, and Equiformer showed reduction in 8 targets with an average of −4.76%. Notably, thermodynamic properties (U_0_, U, H, and G) showed significant performance improvements between −10% and −20% regardless of the baseline network type, while other targets showed improvements between −2% and −5%.

For the MD17 data set using TorchMD-NET as the baseline, error reduction was observed in energy prediction for five molecules, with an average error reduction rate of −7.39% across all eight molecules, as shown in Table 4. In force prediction, seven molecules showed error reduction, except for naphthalene, and the average error change rate was −4.03% when including naphthalene and −13.42% when excluding it.

**Table 3:** MAE of the Predicted Energy and Forces for Four Types of Labels in the xxMD Evaluation^a^

		Energy		Forces	
Molecule type	Label computation	without NP	with NP	without NP	with NP
Azobenzene	CASSCF, s0	0.3646	**0.3631**	**0.0958**	0.0977
	CASSCF, s1	0.4070	**0.3799**	0.1116	**0.1014**
	CASSCF, s2	**0.6061**	0.6173	**0.2340**	0.2412
	DFT	**0.1710**	0.1989	0.1081	**0.0990**
Dithiophene	CASSCF, s0	0.3294	**0.3140**	0.0492	**0.0489**
	CASSCF, s1	**0.2188**	0.2196	0.0483	**0.0482**
	CASSCF, s2	0.2547	**0.2424**	0.0594	**0.0554**
	DFT	0.1293	**0.1103**	0.0787	**0.0725**
Malonaldehyde	CASSCF, s0	0.4480	**0.4049**	**0.1395**	0.1554
	CASSCF, s1	0.4470	**0.4325**	0.2082	**0.1923**
	CASSCF, s2	**0.5594**	0.5701	0.3000	**0.2869**
	DFT	0.1446	**0.1332**	0.1815	**0.1742**
Stilbene	CASSCF, s0	0.4823	**0.4649**	0.1172	**0.1161**
	CASSCF, s1	0.3589	**0.3055**	0.0732	**0.0693**
	CASSCF, s2	0.6114	**0.5658**	0.1668	**0.1529**
	DFT	0.4132	**0.3555**	0.1522	**0.1507**

a TorchMD-NET was selected as the baseline model.

**Table 4:** MAE of the Energy and Forces Prediction on the MD17 Data Set^a^

		TorchMD-NET			
Molecule	Target	w/o NP	w/o NP (2× hidden dim)	w/NP	avg. Δ%
Aspirin	Energy	**0.123**	0.126	0.126	2.38%
	Forces	0.253	0.241	**0.224**	**–12.95%**
Benzene	Energy	0.058	Not converged	**0.05424**	**–6.93%**
	Forces	0.196	Not converged	**0.1174**	**–10.48%**
Ethanol	Energy	**0.052**	0.053	0.0524	0.76%
	Forces	0.109	0.108	**0.0878**	**–24.15%**
Malonaldehyde	Energy	**0.077**	0.097	0.0794	3.02%
	Forces	0.169	0.171	**0.146**	**–15.75%**
Naphthalene	Energy	0.085	0.709	**0.081**	**–4.94%**
	Forces	**0.061**	1.033	0.1594	61.73%
Salicylic acid	Energy	0.093	0.093	**0.08086**	**–15.01%**
	Forces	0.129	0.131	**0.1262**	**–2.22%**
Toluene	Energy	0.074	0.075	**0.058**	**–27.59%**
	Forces	0.067	0.069	**0.057**	**–17.54%**
Uracil	Energy	0.095	0.095	**0.0857**	**–10.85%**
	Forces	0.095	0.089	**0.0857**	**–10.85%**
All molecules	Energy				**–7.39%**
	Forces				^b^ **–4.03%**

a TorchMD-NET was selected as the baseline model.

b The performance gain is −13.42%, except for the case of naphthalene.

To address concerns about whether performance improvements stem from increased computational cost rather than methodological innovation, we conducted additional experiments with double hidden dimensions in the baseline TorchMD-NET model. As shown in the highlighted column in Table 4, simply doubling the hidden dimensions resulted in comparable or worse performance across most molecules compared to the original baseline. Notably, the doubled hidden dimension baseline failed to converge for benzene and showed significantly degraded performance for naphthalene (energy: 0.709 vs 0.085, forces: 1.033 vs 0.061). These results demonstrate that NP’s performance gains cannot be attributed solely to increased parameter count but rather to the effectiveness of the virtual atom representation in capturing molecular interactions.

### Property Prediction in Excited-State Molecules

The experimental results on the xxMD data set demonstrated that NP can also improve the prediction performance for molecules in excited states, which are molecules not in the ground state. We evaluated all molecules, metrics, and their labels calculated from different levels of theory in the xxMD data set, as shown in Table 3. Although both methods showed comparable prediction performances in the case of azobenzene, NP-enhanced TorchMD-NET outperformed in the other three molecular cases: dithiphene, malonaldehyde, and stilbene.

Note that all of the molecules used in this experiment have conjugated systems. Therefore, the performance gain in the xxMD data set may also be attributed to incorporating features distant from atoms, which is similar case to the electron density prediction task introduced above.

### Electron Density Prediction

To validate our method’s effectiveness across both standard molecular properties and complex volumetric data, such as electron density, we evaluated it using the QM9 electron density data set.^26^ We followed the evaluation protocol described in the original paper.^35^

In Table 5, we present the performance comparison of these ablation models, the baseline model, and our NP-enhanced approach on the DeepDFT electron density prediction task. As shown in the table, our NP-enhanced model demonstrated superior performance compared to the baseline on both the relative mean absolute error (ϵ_*mae*_) and the relative root mean squared error (ϵ_*rmse*_). Specifically, it achieved an 11.6% reduction in ϵ_*rmse*_ and a 7.0% reduction in ϵ_*mae*_ compared with the baseline PaiNN model.

**Table 5:** Test Accuracy of Models on the QM9 Electron Density Data Set^a^

Method	Construction of VP (x̃_*t*_)	ϵ_*rmse*_%	ϵ_*mae*_%
DeepDFT	None	0.772	0.270
+ M1	**x**_0_	1.552	0.442
+ M2	x*	0.747	0.273
+ M3	$x_{0}+\frac{t}{T}\left(x^{*}-x_{0}\right)$	0.729	0.267
+ NP	π_*t*_(**ṽ**_*t*_) + **x̃**_*t* ‑ 1_	**0.682**	**0.251**

a DeepDFT, an architecture based on PaiNN, was selected as the baseline model. ϵ_rmse_% and ϵ_mae_% are the mean absolute error (MAE) and root mean square error (RMSE) divided by the total electron density.

Furthermore, to evaluate the motivation behind our methodology, we designed and analyzed three ablation models as follows:

Atom-centered VP (M1): VPs are fixed at the center of each atom (**x̃**_*t*_ = **x**_0_). This configuration tests the necessity of positioning VPs distinctly from atoms.
Static offset VP (M2): VPs are fixed at a precalculated equivariant location denoted as x* (**x̃**_*t*_ = x*). We manually selected a location for x* that maintains equivariance: $x_{i}^{*}=x_{i}+\Sigma_{j\in N(x_{i})}\left(x_{i}-x_{j}\right)$ . This configuration tests a nonlearnable VP positioning strategy.
Moving offset VP (M3): The positions of VPs were iteratively updated toward the fixed offset x* by each layer $\left({\mathrm{x̃}}_{t}=x_{0}+\frac{t}{T}\left(x^{*}-x_{0}\right)\right)$ . This configuration incorporates layer-specific updates but uses nontrainable target positions, enabling comparison against M2 and our fully learnable NP approach.

As a result, the atom-centered VP (M1) showed a significant increase in error compared with the baseline, highlighting the importance of positioning VPs distinctly from atomic nuclei. As indicated in Table 5, using the static offset VP (M2) resulted in an ϵ_*rmse*_ reduction of only 3.3% relative to the baseline. Employing the moving offset VP (M3) yielded a slightly better reduction of 5.6%. This suggests a minor benefit from layer-specific updates even with a nonlearnable target. However, both improvements (3.3% and 5.6%) were substantially smaller than the 11.6% reduction achieved by our NP approach, and both M2 and M3 resulted in errors considerably higher than those of the proposed NP method. These outcomes strongly suggest the significance of the learnable projection function π_*t*_ used in our NP approach, which learns to optimally position the VPs, compared to the nonlearnable strategies of M2 and M3.

In summary, these experiments demonstrate that both positioning VPs distinctly from atoms and computing their locations in a learnable manner are key factors underpinning the efficacy of the proposed NP method, whereas layer-specific updates appear to play a secondary role.

### Case Study on DeepDFT Data Set

To investigate how neural polarization (NP) enhances model performance, we conducted case studies investigating its impact on the DeepDFT data set, which spans a variety of molecular species. We evaluated prediction errors against the ground-truth electron density for the baseline model and the NP-enhanced model, and representative cases are visualized in Figure 3 (additional examples are provided in Appendix A). Specifically, the figure depicts (a) the ground-truth electron density distribution, (b) the baseline model’s error map, (c) the NP-enhanced model’s error map, and (d) the relative error reduction achieved by the NP enhancement.

**Figure 3:**
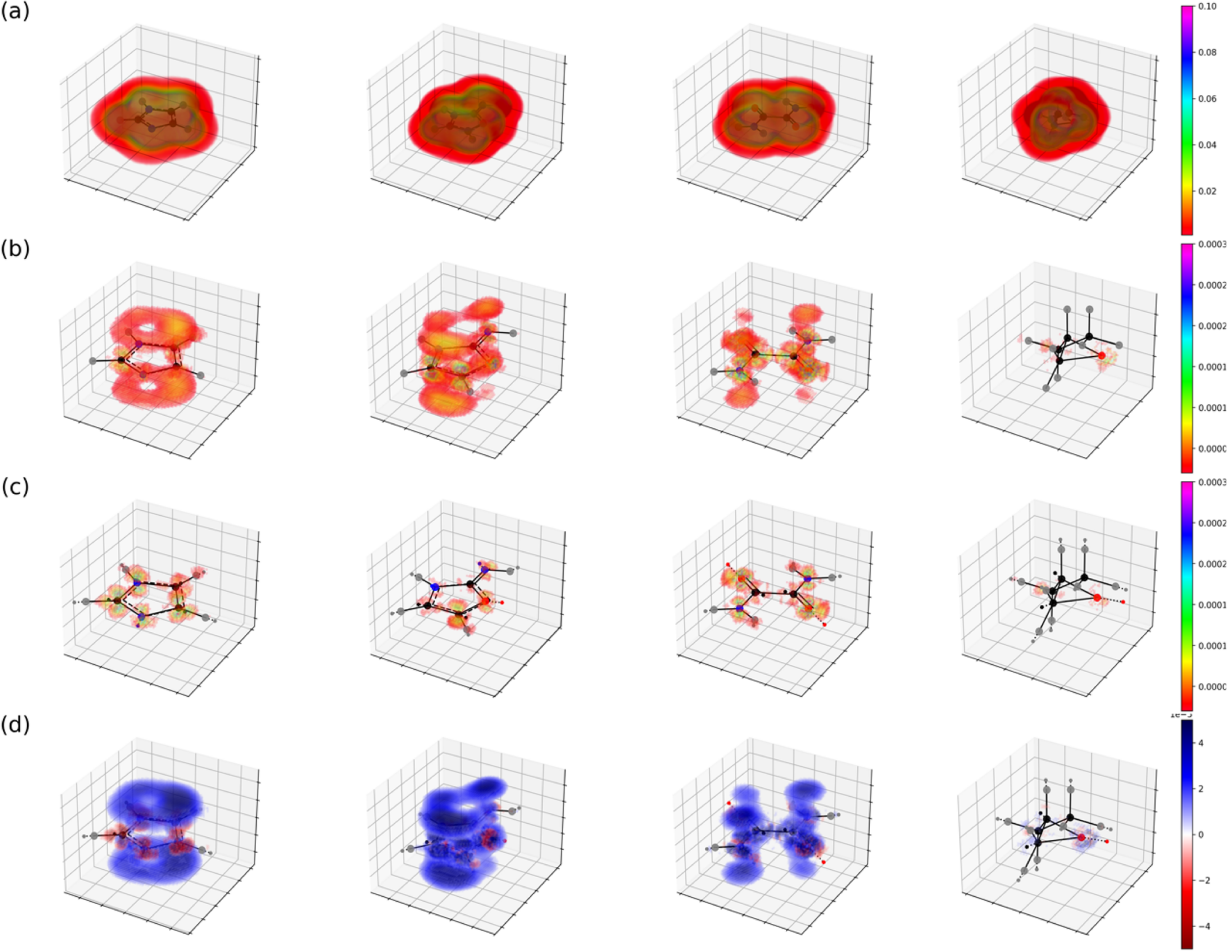
Point-cloud visualization of electron density predictions: (a) Ground-truth electron density from the QM9 data set. (b) Prediction errors from the original DeepDFT model. (c) Prediction errors from the NP-enhanced model. (d) Visualization of error reduction, highlighting significant improvements in delocalized electron regions of the NP-enhanced model. Each VP (*x̃*_*i*_) is shown as a small dot connected with a gray line attached to each atom in figures with NP (c and d).

Our results, as illustrated by these case studies, indicate that the NP-enhanced model achieves noticeably improved accuracy in regions characterized by electron delocalization, particularly in the electron density lobes situated above and below the molecular plane of the conjugated systems shown in (a). In these regions, the baseline model exhibits significant deviations (b), whereas the NP-enhanced model shows fewer errors (c), as quantified by the relative error reduction (d).

The observed specificity of the error reduction in delocalized regions suggests the potential of the NP mechanism in molecules with polarization effects. This characteristic could be a factor contributing to the overall performance gains observed across diverse molecular tasks.

### Effect of Model Depth on Neural Polarization Performance

To further investigate the efficiency of neural polarization in capturing molecular interactions, we conducted experiments with reduced network depth to assess whether NP-enhanced models can achieve comparable performance with fewer message-passing layers. The objective of this study is to investigate whether neural polarization can achieve comparable performance with fewer message-passing layers through more efficient information propagation facilitated by virtual atoms.

We designed shallow networks with 2 fewer layers for both the baseline TorchMD-NET and NP-enhanced variants: from 6 to 4 layers for MD17 experiments and from 8 to 6 layers for QM9 experiments. The evaluation was performed on four representative molecules from MD17 (aspirin, ethanol, toluene, and uracil) and the U_0_ property prediction task from QM9 (Table S2).

The results demonstrate that NP-enhanced models maintain competitive performance, even with reduced network depth. Most importantly, NP (Shallow) models consistently perform comparably to or better than the original baseline models across most prediction tasks despite using fewer layers. For example, NP (shallow) outperforms the original baseline in aspirin energy prediction (0.121 vs 0.123) and maintains superior performance in toluene energy prediction (0.060 vs 0.074). Similar trends are observed across other molecules and prediction targets. Interestingly, for U_0_ in QM9, the MAE showed a decreasing trend in shallow environments regardless of whether NP was applied.

These findings indicate that virtual atoms provide sufficient representational enhancement to achieve competitive performance with fewer message-passing layers compared with conventional architectures. This suggests that neural polarization enables more efficient information propagation pathways, allowing shallower networks to capture molecular interactions effectively.

### Analysis of Challenging Cases

We conducted error analysis on individual samples by evaluating the test set using five different pretrained models (μ, ϵ_HOMO_, *R* ^2^, *U*_0_, and *zpve*) based on the TorchMD-NET architecture trained on the QM9 data set. We observed that certain test set samples consistently produced relatively high error values across all pretrained model types, which we designated as challenging cases. Nine representative examples are presented here. For instance, Case 1 represents an H_2_O molecule that exhibited exceptionally high error values across all five pretrained models (MAE values: μ = 0.892, ϵ_HOMO_ = 0.673, *R* ^2^ = 0.537, and *zpve* = 0.147), while the remaining eight molecules similarly showed less accurate predictions with error values significantly exceeding the respective label averages across multiple prediction tasks. We identified that these challenging cases possess distinctive structural features and found that μ and *R* ^2^ predictions, which generally performed poorly with the TorchMD-NET architecture, produced particularly extreme error values for these samples relative to their overall MAE. To better understand model limitations, we selected nine representative cases with consistently high prediction errors across multiple labels for detailed analysis (Figure 4).

**Figure 4:**
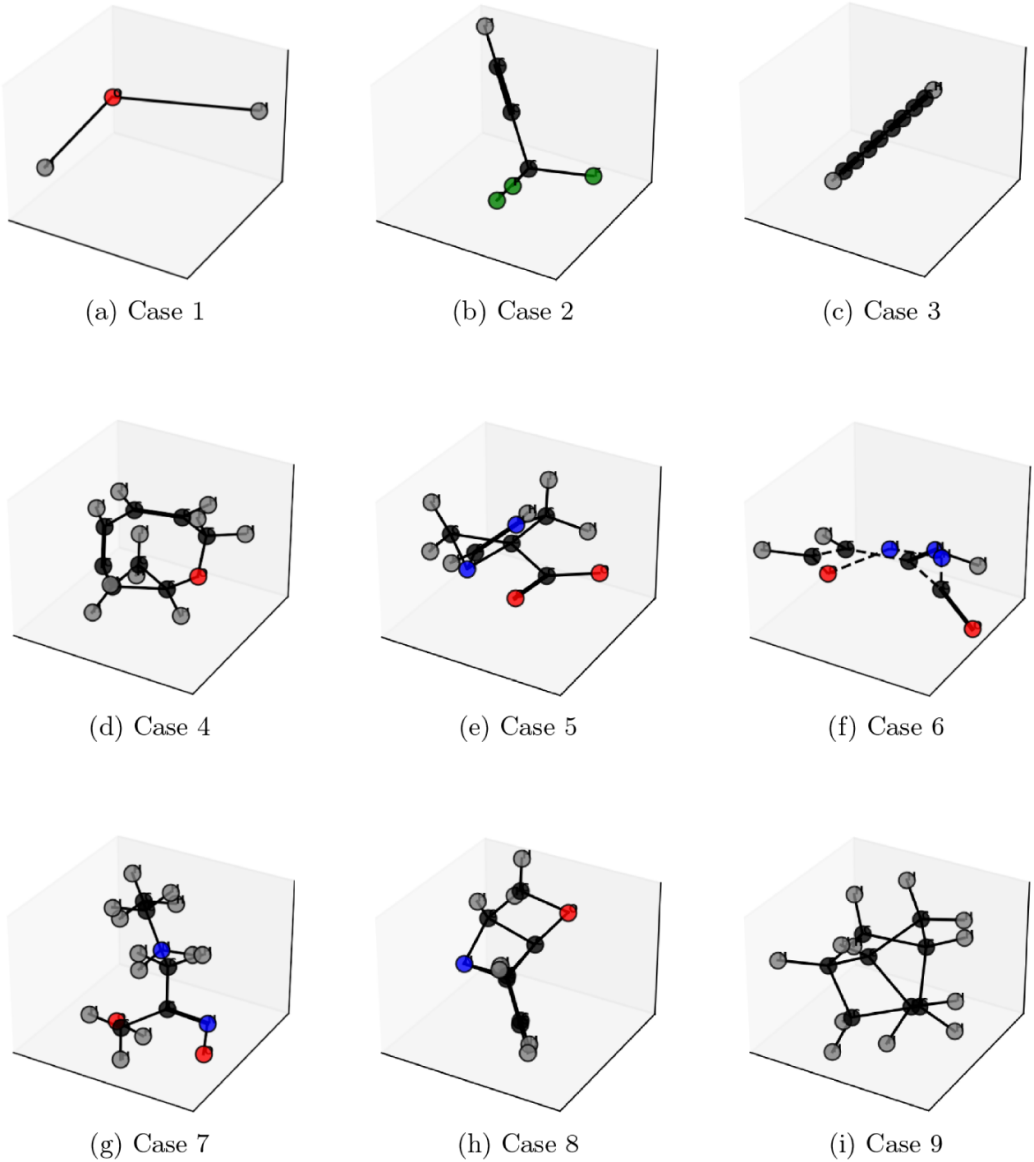
Analysis of molecular structures with high prediction errors. We present a comparison of structural features and error values for selected samples, showing consistently high test errors across multiple labels. Atoms are color coded as follows: carbon (black), oxygen (red), nitrogen (blue), fluorine (green), and hydrogen (gray). Bond types are not distinguished or are displayed separately.

This error analysis revealed key insights into model performance limitations. Labels with poor overall performance (μ and *R* ^2^ cases with TorchMD-NET) were particularly susceptible to errors in certain samples. We identified specific molecular types that consistently produced high errors across multiple prediction tasks, including very small molecules (e.g., H_2_O), elongated geometries, and structures with complex cyclic arrangements. Our findings indicate that elevated average MAE values primarily stemmed from a small subset of challenging samples producing large prediction errors in out-of-distribution cases. These patterns suggest potential areas for future methodological improvements, particularly in handling diverse molecular geometries and optimizing training procedures for structurally complex cases.

## Conclusion

We developed NP, a novel method that expands the molecular representation space by introducing features capable of learning delocalized electron density. NP can be flexibly implemented in any equivariant network, without enforcing any additional regularizers or objective functions. NP effectively addresses the potential limitations of previous models in representing atomic environments, such as electron density. Various ablation studies further confirmed that NP’s advantages do not merely result from duplicating atom representations. Although these findings align with our motivation for NP, further studies may be needed to elucidate the precise mechanistic basis of the observed improvements. Our analysis revealed that some molecular structures, particularly small molecules and complex cyclic configurations, remain challenging to predict, suggesting directions for future improvements to our approach. Furthermore, the increased computational cost due to the growth in the number of nodes and edges poses limitations for applications to large molecules. Although we observed that NP-enhanced architectures can efficiently learn molecular properties with fewer message-passing steps, this high computational complexity issue also needs to be addressed in future work. Based on these results, we expect NP to enforce improvements in general molecular problems.

While NP does not change the computational complexity of the original model, it introduces an additional computational cost. Furthermore, the trajectories obtained from **x̃** of trained models do not seem to provide explicit knowledge about electron density or delocalization, although we observed some directional patterns in **x̃** across the molecules. In future work, we propose various methodologies inspired by aspects beyond polarization.

## Supplementary Material



## Footnotes

1 EGNN: https://github.com/lucidrains/egnn-pytorch; Equiformer: https://github.com/lucidrains/equiformer-pytorch; TorchMD-NET: https://github.com/torchmd/torchmd-net and DeepDFT: https://github.com/peterbjorgensen/DeepDFT.

## Notes

Data sets: detailed descriptions of QM9, MD17, and xxMD data sets including molecular compositions, computational methods, and evaluation protocols. Detailed implementation of neural polarization: comprehensive implementation details for neural polarization across different baseline networks (EGNN, TorchMD-NET, PaiNN, Equiformer), including projection layer configurations, hyperparameter settings, and computational requirements. Ablation study in QM9: performance comparison results demonstrating that neural polarization improvements are not solely due to increased computational costs, with quantitative analysis on ϵ_HOMO_, μ, and *U*_0_ targets (Table S1). Effect of model depth on neural polarization performance: performance comparison of neural polarization with reduced number of layers on MD17 and QM9 data sets, demonstrating efficiency with fewer message-passing steps (Table S2). Examples of training curves: Figure S1: training curves of neural polarization across different data sets, including (a–c) learning curves on MD17 data set for three representative molecules, (d) training progress on QM9 data set, and (e–f) performance curves on DeepDFT data set for two different configurations. Additional figures for QM9 electron density data set: Figure S2: electron density point clouds for molecules in QM9 data set; Figure S3: prediction error point clouds at original model for molecules in QM9 data set; Figure S4: prediction error point clouds at NP-enhanced model for molecules in QM9 data set; Figure S5: error reduction by neural polarization for molecules in QM9 data set (PDF)

## §

LG Chem, Seoul 07795, Republic of Korea

## #

Danggeun Market Inc., Seoul 06611, Republic of Korea
