# Supplementary material for: Introducing Virtual Points in Equivariant Networks by Extending Atom Representation for Effective Prediction
Source: J Chem Theory Comput. 2025 Aug 25;21(17):8468–77. doi: 10.1021/acs.jctc.5c00701 (PMC12424937; doi:10.1021/acs.jctc.5c00701)
Supplement: Supplementary file 1 [file ct5c00701_si_001.pdf]

Introducing virtual points in equivariant networks by extending atom representation for effective prediction

Bumju Kwak[†] and Jeonghee Jo[*,‡]

[†]*Independent researcher, Danggeun Market Inc., Seoul 06611, Republic of Korea*

[‡]*Independent researcher, LG Chem, Seoul 07795, Republic of Korea*

E-mail: jade.jeonghee.jo@gmail.com

Supporting Information Available

Datasets

The QM9 dataset[1] is a dataset containing various molecular properties for 134k small molecules. The molecules included in the dataset are composed of C, N, O, H, and F and contain up to 29 atoms. This dataset is frequently used for measuring the performance of deep learning models for molecules. The dataset consists of molecular conformations, the atomic coordinates of the given molecule, and 12 different molecular properties calculated from these conformations, such as internal energy and dipole moment.

A dataset of electron densities for QM9 molecules was presented by.[2] The dataset consists of electron densities evaluated at three-dimensional grid points for all molecules within the QM9 dataset. The computation of electron density was obtained by KS-DFT with the PBE exchange-correlation functional.The dataset split and evaluation criteria in the same way as in.[2] We note that we excluded one data in the test set because of an extraordinary large error was produced from this datapoint in both the original DeepDFT and NP-enhanced DeepDFT, which may be caused by an unknown issue.

The MD17 dataset[3,4] is a dataset about molecular dynamics (MD) trajectories of 10 small molecules. The dataset consists of molecular conformations from the trajectories with the corresponding molecular energy and atomic forces. The task for this dataset is to predict the forces and energy for a given conformation. In this paper, we used the MD17 dataset configuration addressed in TorchMD-NET, which is the base model for comparison.

The xxMD dataset[5] was developed for the extended excited-state molecular dynamics, consisting of four types of molecules sampled from nonadiabatic dynamics trajectories. Specifically, xxMD is composed of four molecules: azobenzene, stilbene, malonaldehyde, and dithiophene. The potential energies and forces of each conformation were calculated by two types of different computational methods, xxMD-CASSCF and xxMD-DFT, respectively. In xxMD-CASSCF, the energy and forces were calculated by the first three singlet electronic

states (S0, S1, and S2) at the state-averaged complete active state self-consistent field (SA-CASSCF) electronic theory, which can provide more complicated potential energy surface (PES). The other dataset, xxMD-DFT was calculated by a single-point spin-polarized Kohn-sham DFT (KS-DFT)[6] on the same geometries of xxMD-CASSCF dataset. The evaluation metrics are MAE of energy and forces.

Although the MD17 dataset was limited in relatively narrow region of PES close to the equilibrium state, xxMD dataset include more diverse conformations or out-of-distribution cases, especially bond breaking and formation. This diversity enables us to explore more out-of-distribution but significant cases in chemical reactions.

For the xxMD experiments, we also used TorchMD-NET[7] as the baseline framework. The learning rate was set in the range of 1e-4 to 5e-4. All other hyperparameters were kept identical to those used in the MD17 experiments of the original TorchMD-NET. All data splits followed the protocol provided in the original dataset paper. Additional details regarding the dataset statistics can be found in the original paper.

Detailed Implementation of Neural Polarization

We implemented NP based on the official GitHub repositories provided by each model. NP was implemented using the same way with three types of baseline networks, except for the projection layer. For EGNN[8] we used Multilayer Perceptron with a single hidden layer of 128 dimension as a node feature. In TorchMD-NET[7] and PaiNN,[9] the projection layer was implemented as a single linear layer without bias for the $l = 1$ feature of each layer output $\tilde{\mathbf{v}}_{\mathbf{t},\mathbf{i}}$. For Equiformer,[10] the projection layer was implemented as a shallow equivariant network based on a tensor product with one $l = 1$ feature.

To minimize undesired effect on performances arose by hyperparameter optimization, we fixed all hyperparameters except the learning rate and the batch size provided in the official repositories. We modified batch size as half only in cases where out-of-memory errors occurred. For other implementations such as data splitting, optimizer, learning rate scheduling

strategy, and objective loss function, we followed configurations implemented in the official repositories. To mitigate gradient exploding issues at short interatomic distances in the PaiNN model when applying NP, we implemented a lower bound of 0.01 nm on the computed interatomic distances. Since the original PaiNN model inherently maintains interatomic distances above this threshold, this clipping operation is not expected to impact the model's existing functionality.

Experiments were conducted on NVIDIA V100, A40, or A100 GPUs. Each experiment with the QM9 dataset has required maximum 240 GPU hours each, while other experiments required less than 24 GPU hours. Training times differ from model types, since we followed the early-stopping condition implemented in each original repository.

Ablation study in QM9

We conducted an ablation study to examine whether the improved performance in molecular property prediction was simply due to increased computational costs. We tested the M1 strategy on ϵ_{HOMO}, μ, and U_0, and as Table S1 indicates, this approach actually led to higher prediction errors. These findings demonstrate the effectiveness of neural polarization (NP), showing performance improvements that are not solely explained by increased computational resources. Furthermore, the results confirm that updating VP positions using model parameters enhances the prediction performance.

Table S1: Ablation results trained with Equiformer on three types of QM9 targets μ, ϵ_{HOMO} and U_0. $\{\mathbf{x}, \mathbf{x}\}$ in the second row is the ablation study for comparing the effect of $\tilde{\mathbf{x}}$, keeping the same computational cost and weight parameters. Scale is the same with the original.

Method	μ	ϵ_{HOMO}	U_0
Equiformer	0.0118	**16.5**	6.59
+ M1	0.0159	17.7	8.80
+ NP	**0.0109**	16.7	**5.54**

Effect of Model Depth on Neural Polarization Performance

Table S2: Performance comparison of Neural Polarization with reduced number of layers on MD17 and QM9 dataset. Both baseline TorchMD-NET and TorchMD-NET w/NP were tested with reduced layers (6→4 for MD17 and 8→6 for QM9, respectively), labeled as 'Shallow'. The units of energy and forces for the MD17 dataset are kcal/mol and kcal/mol/Å, respectively, while the U_0 for the QM9 dataset is meV.

Molecule	Target	TorchMD-NET (MAE)			
		w/o NP	w/o NP (Shallow)	w/ NP	w/ NP (Shallow)
MD17 (Aspirin)	Energy	0.123	0.128	0.126	0.121
	Forces	0.253	0.273	0.224	0.258
MD17 (Ethanol)	Energy	0.052	0.054	0.052	0.052
	Forces	0.109	0.116	0.088	0.108
MD17 (Toluene)	Energy	0.074	0.075	0.058	0.060
	Forces	0.067	0.068	0.057	0.063
MD17 (Uracil)	Energy	0.095	0.095	0.086	0.091
	Forces	0.095	0.096	0.086	0.086
QM9 (U_0)	Energy	6.15	5.8	5.5	5.3

Examples of training curves

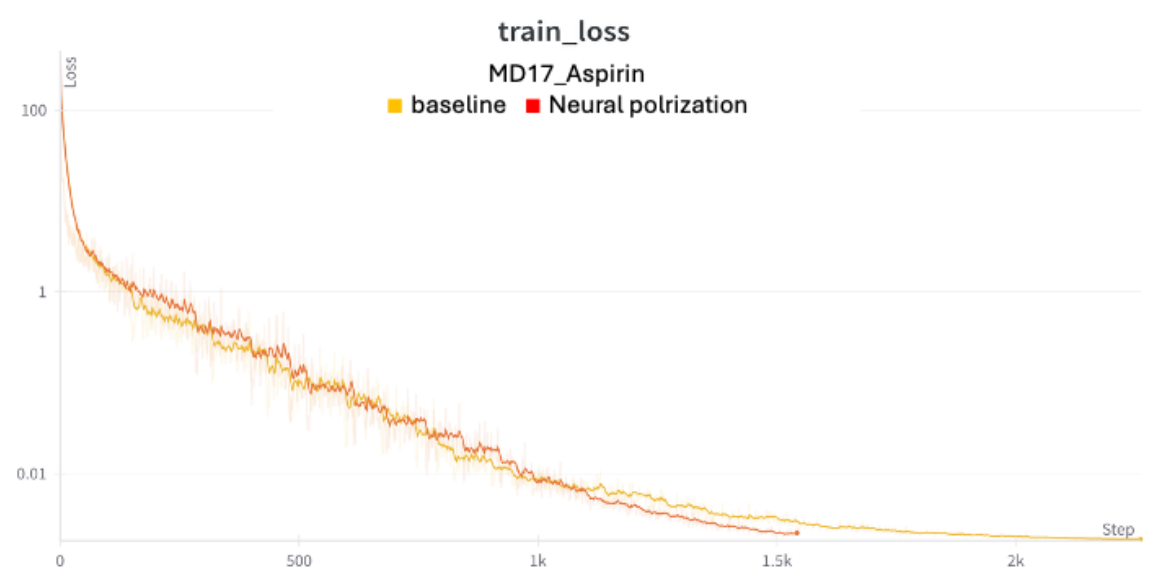

(a) MD17 - Aspirin

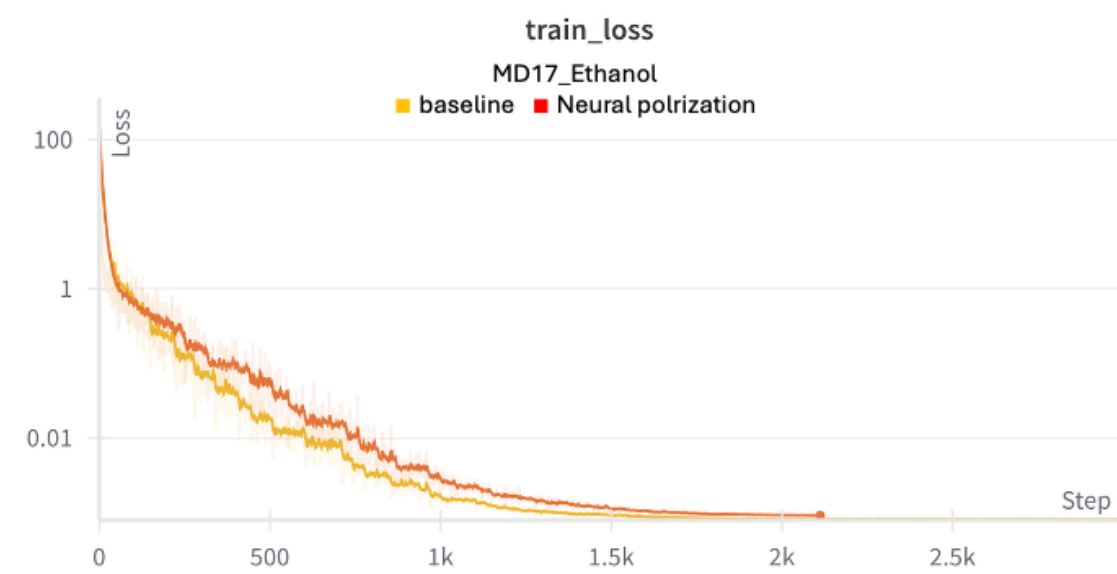

(b) MD17 - Ethanol

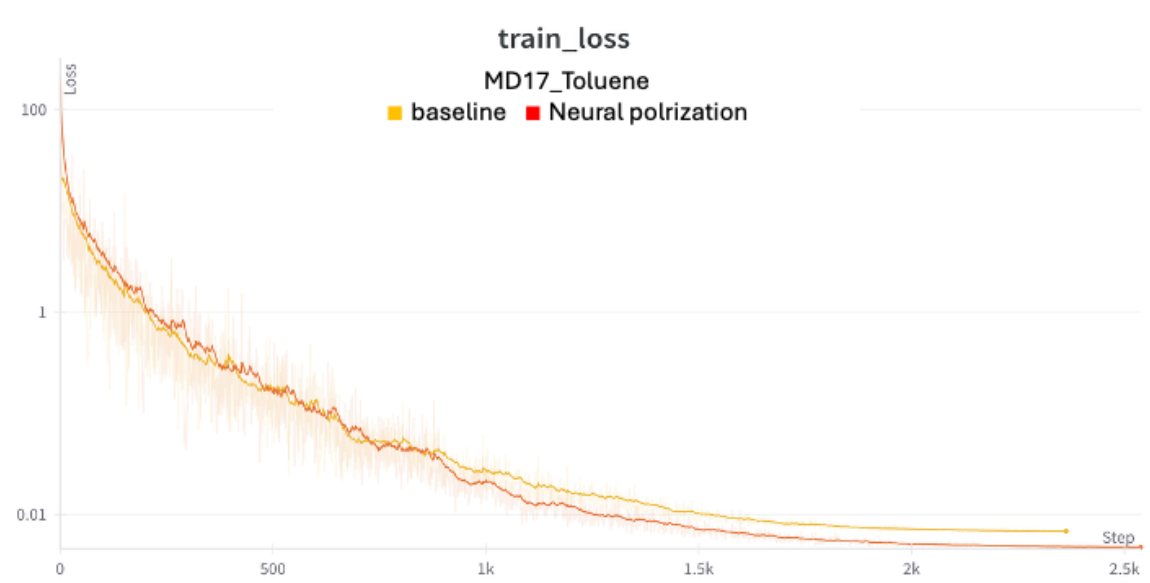

(c) MD17 - Toluene

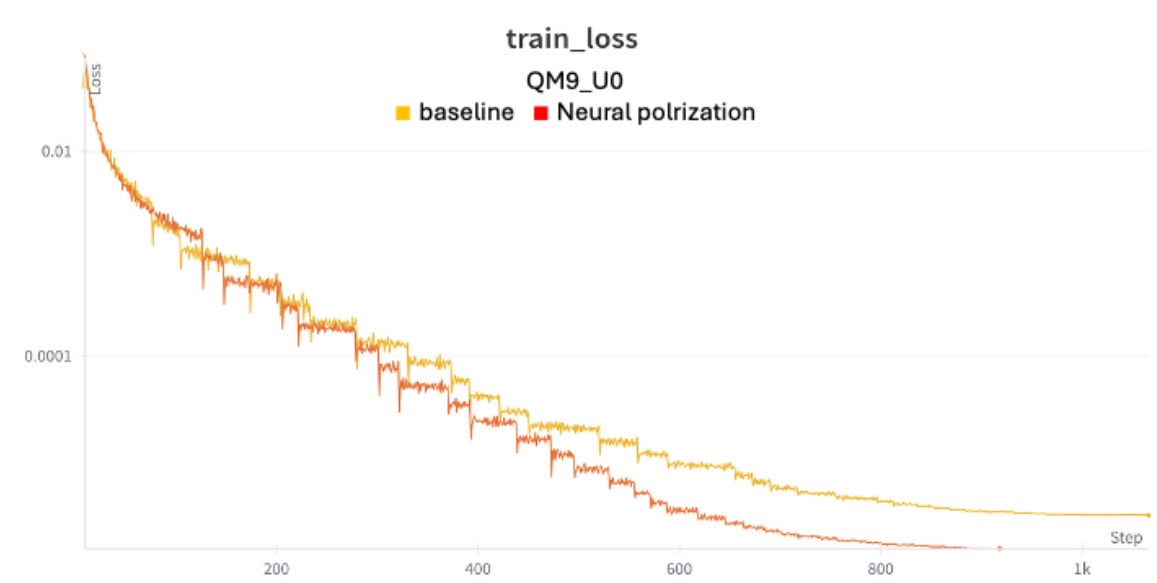

(d) QM9 - U0

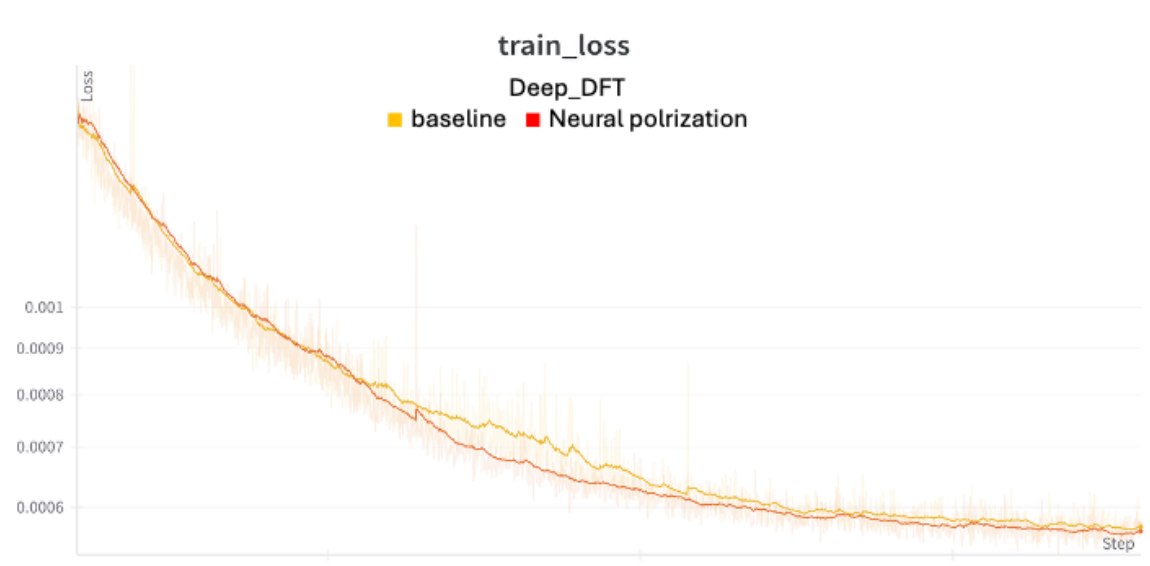

(e) DeepDFT

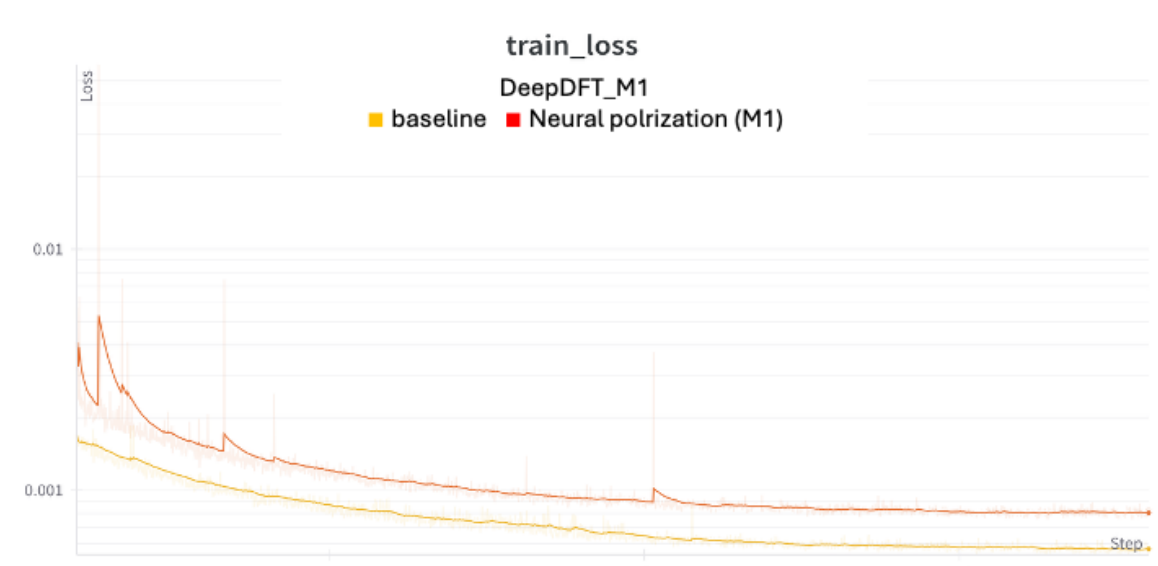

(f) DeepDFT (M1 setting for comparison)

Figure S1: Training curves of Neural Polarization across different datasets. (a-c) Learning curves on MD17 dataset for three representative molecules. (d) Training progress on QM9 dataset. (e-f) Performance curves on DeepDFT dataset for two different configurations.

Additional Figures for QM9 electron density dataset

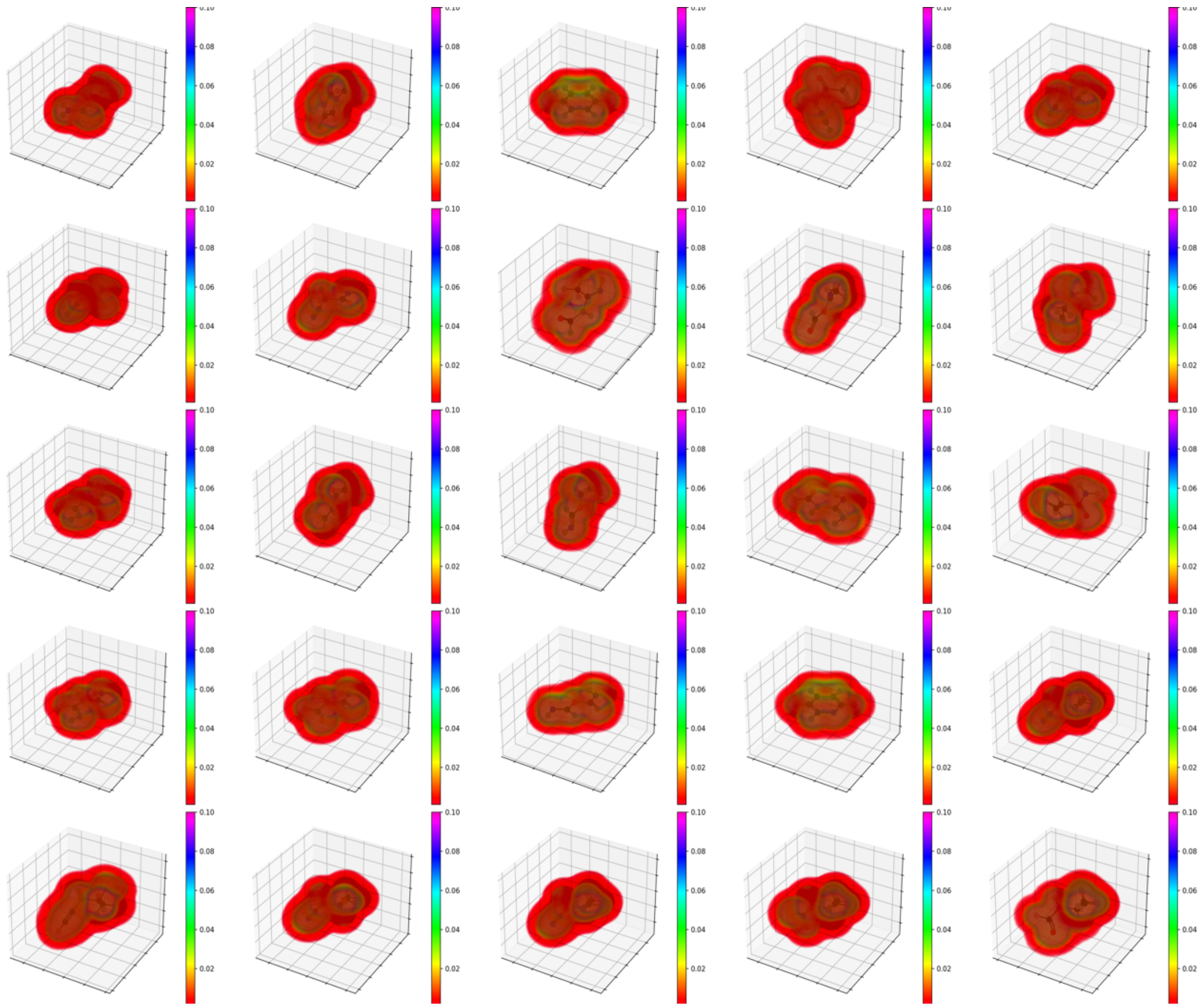

Figure S2: Electron density point clouds for molecules in QM9 dataset.

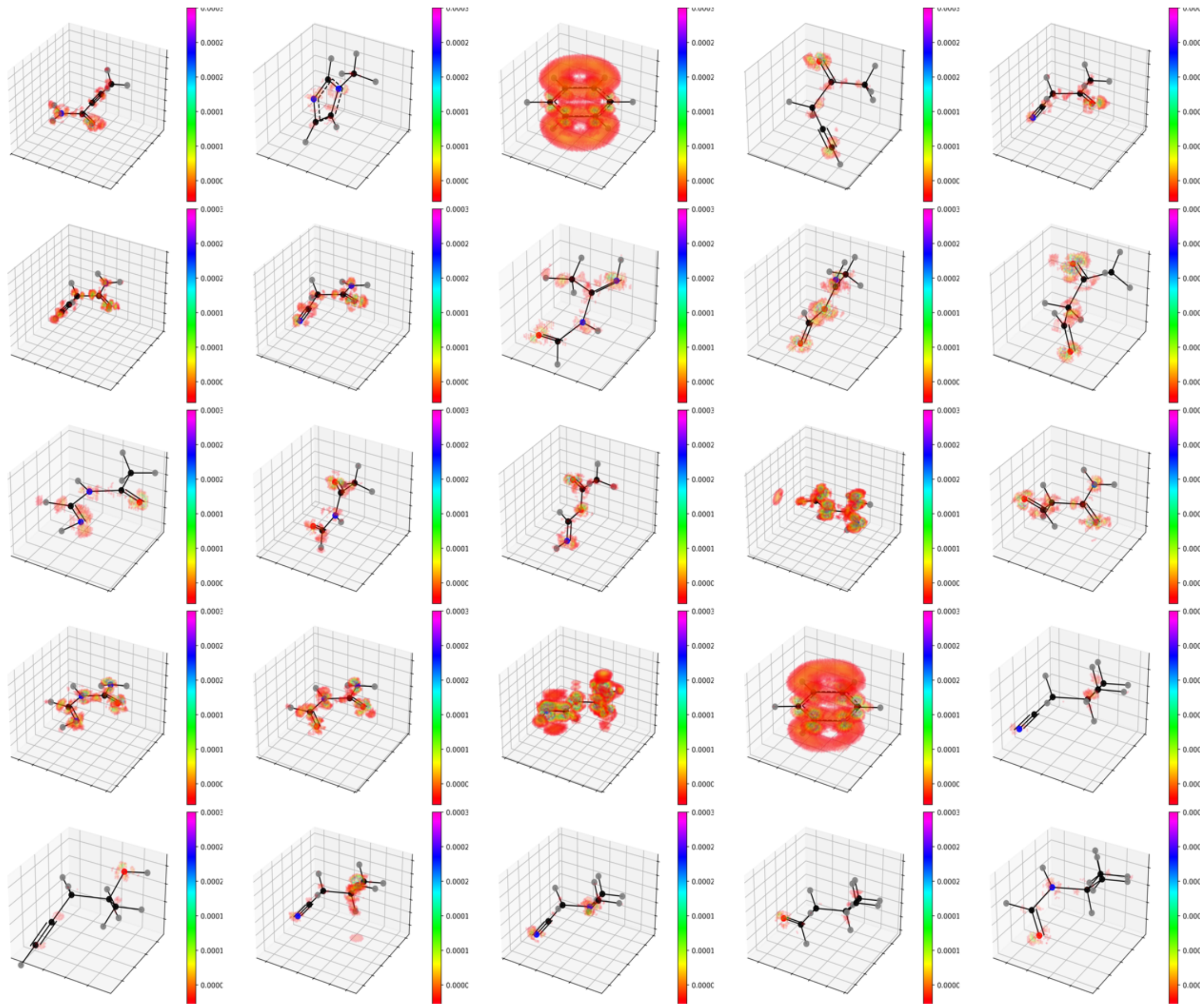

Figure S3: Prediction error point clouds at original model for molecules in QM9 dataset.

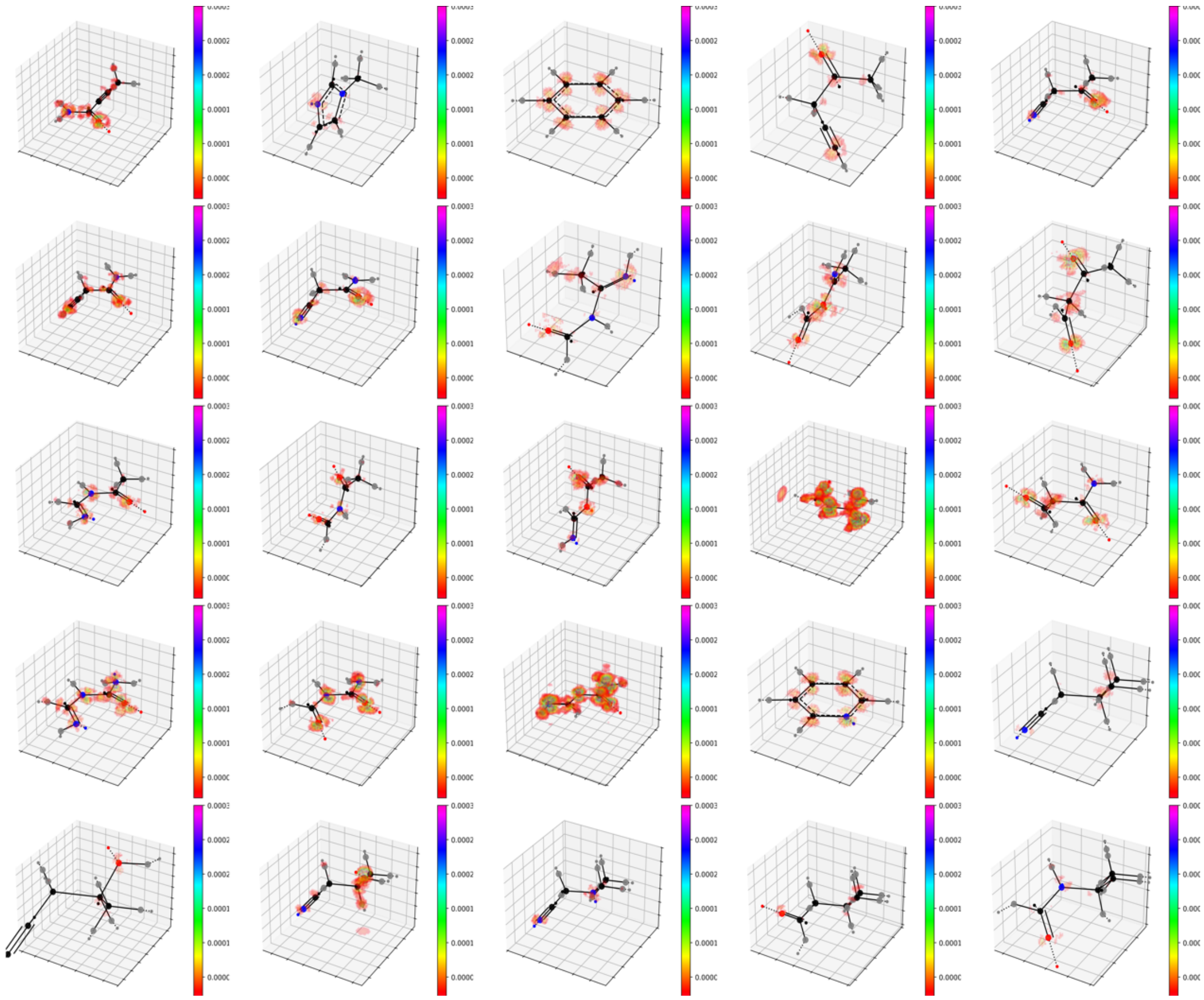

Figure S4: Prediction error point clouds at NP-enhanced model for molecules in QM9 dataset.

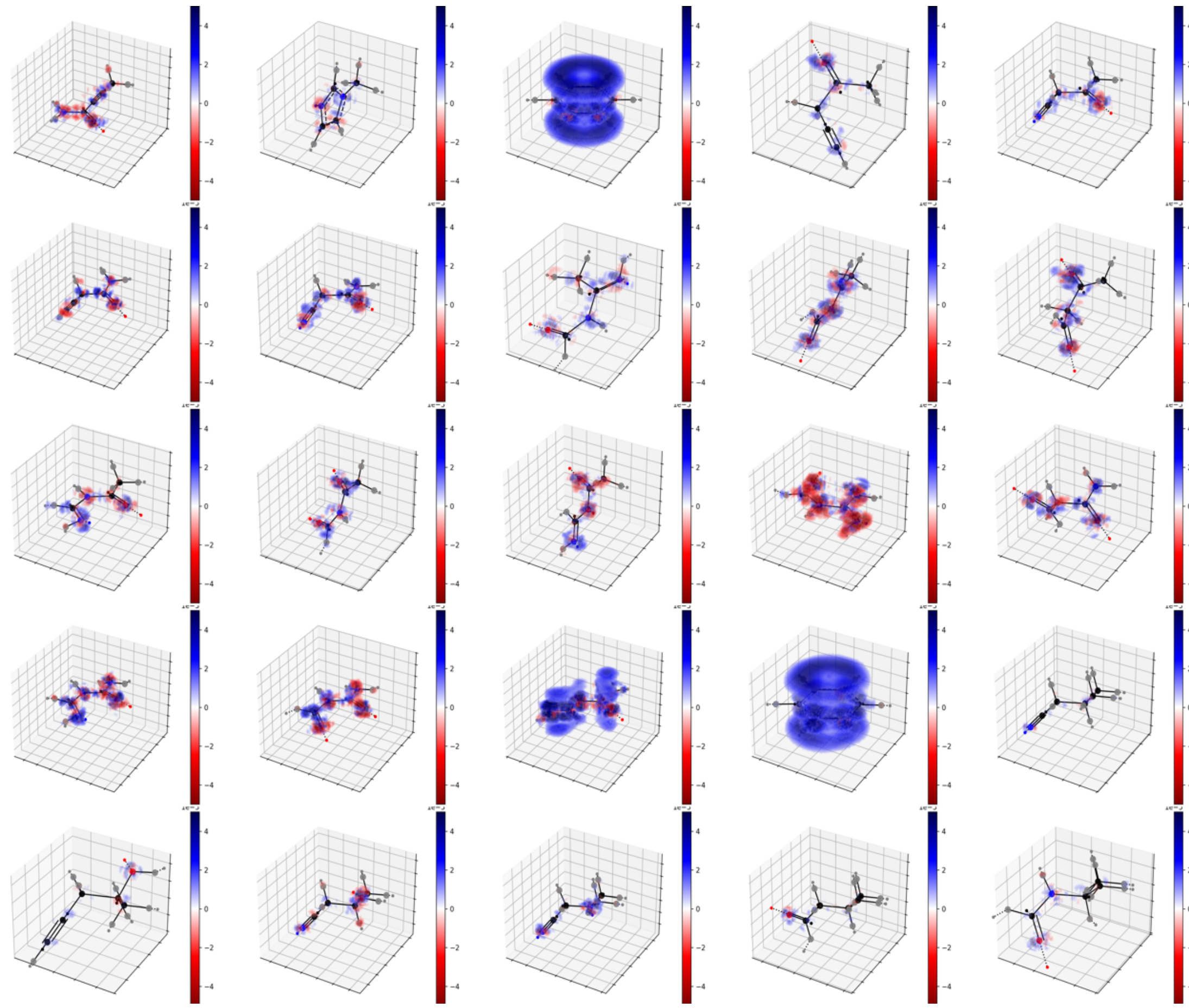

Figure S5: Error Reduction by NP for molecules in QM9 dataset.

References

(1) Ramakrishnan, R.; Dral, P. O.; Rupp, M.; von Lilienfeld, O. A. Quantum chemistry structures and properties of 134 kilo molecules. *Scientific Data* **2014**, *1*.

(2) Jørgensen, P. B.; Bhowmik, A. DeepDFT: Neural Message Passing Network for Accurate Charge Density Prediction. Machine Learning for Molecules Workshop@ NeurIPS 2020. 2020.

(3) Chmiela, S.; Tkatchenko, A.; Sauceda, H. E.; Poltavsky, I.; Schütt, K. T.; Müller, K.-R. Machine learning of accurate energy-conserving molecular force fields. *Science advances* **2017**, *3*, e1603015.

(4) Christensen, A. S.; Von Lilienfeld, O. A. On the role of gradients for machine learning of molecular energies and forces. *Machine Learning: Science and Technology* **2020**, *1*, 045018.

(5) Pengmei, Z.; Liu, J.; Shu, Y. Beyond MD17: the reactive xxMD dataset. *Scientific Data* **2024**, *11*, 222.

(6) Kohn, W.; Sham, L. J. Self-consistent equations including exchange and correlation effects. *Physical review* **1965**, *140*, A1133.

(7) Thölke, P.; De Fabritiis, G. Torchmd-net: Equivariant transformers for neural network based molecular potentials. *arXiv preprint arXiv:2202.02541* **2022**,

(8) Satorras, V. G.; Hoogeboom, E.; Welling, M. E(n) equivariant graph neural networks. International conference on machine learning. 2021; pp 9323–9332.

(9) Schütt, K.; Unke, O.; Gastegger, M. Equivariant message passing for the prediction of tensorial properties and molecular spectra. International Conference on Machine Learning. 2021; pp 9377–9388.

(10) Liao, Y.-L.; Smidt, T. Equiformer: Equivariant Graph Attention Transformer for 3D Atomistic Graphs. The Eleventh International Conference on Learning Representations. 2022.
